# Mean platelet volume and neutrophil to lymphocyte ratio in patients with tinnitus: a case-control study

**DOI:** 10.1016/j.bjorl.2020.05.006

**Published:** 2020-06-04

**Authors:** Selçuk Yildiz, Harun Karaca, Sema Zer Toros

**Affiliations:** a Haydarpaşa Numune Training and Research Hospital, Department of Otorhinolaryngology, Head and Neck Surgery, Istanbul, Turkey; b Karakoçan State Hospital, Department of Otorhinolaryngology, Head and Neck Surgery, Elazig, Turkey

**Keywords:** Tinnitus, Mean platelet volume, Neutrophil to lymphocytes ratio

## Abstract

**Introduction:**

Different theories have been proposed on the etiology of tinnitus, including metabolic and audiologic causes. We suggest that mean platelet volume and neutrophil to lymphocyte ratio levels change in tinnitus, indicating microcirculatory disturbance and inflammatory process in the etiopathogenesis of tinnitus.

**Objectives:**

We aimed to evaluate the mean platelet volume and neutrophil to lymphocyte ratio in patients with tinnitus in comparison to healthy controls.

**Methods:**

Retrospective case-control study. Two-hundred and eighty-seven patients aged 18–59 years and diagnosed with tinnitus in the Ear, Nose, and Throat Clinic between December 2014 and May 2017 (patient group) and 275 healthy individuals who applied for a hearing screening within the same time period (control group). Demographics, concomitant diseases, laboratory results, and audiometric data were recorded. Mean platelet volume and neutrophil to lymphocyte ratio were the outcome measures. Patients with hearing loss due to presbycusis or another reasons, and patients with anatomical disorders in the external and middle ear were excluded from the study by using physical examinations, pure audio audiometry results and radiological imaging. The upper age limit was set at 59 to exclude presbycusis patients.

**Results:**

The ratio of female patients was higher in patient group than control group (58.5%, *n* = 168 vs. 49.4%, *n* = 127; respectively; *p* = 0.033). The mean age of patient group was significantly higher than those of control group (44.89 ± 10.96 years and 38.37 ± 10.65 years, respectively; *p* = 0.001). The percentage of subjects with high mean platelet volume level was significantly higher in patient group than control group (9.4%, *n* = 27, and 3.1%, *n* = 8 respectively; *p* = 0.008). The mean neutrophil to lymphocyte ratio was higher in patients with tinnitus than control group (1.95 ± 1.02 and 1.67 ± 0.57, *p* = 0.012). A neutrophil to lymphocyte ratio level of 2.17 and above is associated with 1.991 times higher risk of tinnitus (odds ratio = 1.99, 95% confidence interval 1.31–3.02).

**Conclusion:**

High mean platelet volume and neutrophil to lymphocyte ratio values are associated with idiopathic tinnitus, suggesting the role of vascular pathologies in etiology of tinnitus. Tinnitus may be a sign of underlying systemic or local disorders. Therefore, patients with tinnitus should undergo detailed evaluation including hematological indices.

## Introduction

Tinnitus is the perception of abnormal sound such as fast flow, buzzing, whistling, ringing, and humming in the ears. It is a common condition affecting about one fifth of the general population, and thus constitutes a significant reason for admission to Ear, Nose, and Throat (ENT) clinics.^1^, ^2^, ^3^ Since its treatment is directed to the underlying cause, the etiology of tinnitus should be thoroughly investigated.

Different theories have been proposed on the etiology of tinnitus, including metabolic and audiologic causes. One of the theories has suggested that a damage in pathological synapses in neighboring nerve fibers is the underlying cause of tinnitus.^4^, ^5^ Another theory proposed that any damage in inner or outer hair cells leads to tinnitus by increasing spontaneous activity.^4^, ^5^ Since the inner ear has a blood supply without collaterals, microcirculatory disturbances have been also suggested to play a role in the damage causing tinnitus. In recent years, the mean platelet volume (MPV) and neutrophil to lymphocyte ratio (NLR) have been commonly studied to investigate the etiopathogenesis and prognosis of microvascular diseases.^6^, ^7^, ^8^ It has been suggested that risk of thrombosis increases as the size of the thrombocytes grows in microvascular diseases.^6^, ^7^, ^8^ Therefore, high MPV has been associated with high incidence of microvascular complications in diabetes mellitus and increased mortality in coronary artery disease.^9^, ^10^ NLR has been considered as an indicator of inflammatory stress and used as a marker of systemic inflammatory status.^11^ It has been reported that increased NLR values are correlated with high mortality in patients with coronary artery disease.^12^ Based on this information, we suggest that MPV and NLR levels in tinnitus indicate microcirculatory disturbance and inflammatory process in the etiopathogenesis of tinnitus.

Therefore, in this study, we aimed to evaluate MPV and NLR levels in patients with tinnitus in comparison to healthy controls.

## Methods

### Study design and population

Two-hundred and eighty-seven patients aged 18–59 years and diagnosed with tinnitus in the ENT clinic between December 2014 and May 2017 (patient group) and 257 healthy individuals who applied for a hearing screening within the same time period (control group) were included in this retrospective case-control study. Patients who had anatomical disorders of external and middle ear that are determined by physical examination findings and computed tomography scans, history of ear surgery, hearing loss, and those with pure tone averages higher than 20 dB on audiogram were excluded from the study. The upper age limit was set at 59 to exclude presbycusis patients.

The study was approved by the Institutional Clinical Research Ethics Committee, and conducted in accordance to the ethical principles for medical research involving human subjects outlined in the Helsinki Declaration (Approval number of the ethics committee: 2017/67). Informed consent requirement was waived due to the retrospective design of the study.

Demographics, medical history, concomitant diseases, physical examination findings, laboratory results, and audiometric data were recorded, and statistically compared between patients and control groups.

The normal MPV range in complete blood was accepted as 4.5–8.5 femtoliter (fL).^13^ The neutrophil and lymphocyte counts were used to calculate NLR.

Audiometric data were analyzed using seven-frequency pure-tone averages (250 Hz, 500 Hz, 1000 Hz, 2000 Hz, 4000 Hz, 6000 Hz, and 8000 Hz) of air and bone conduction for both ears.

### Statistical analysis

The study data were summarized by using descriptive statistics (e.g., mean, standard deviation, median, range, frequency, percentage). The Number Cruncher Statistical System software (NCSS, 2007; Kaysville, Utah, USA) was used for the statistical analysis. In order to test the significance of difference in continuous variables between study groups, Student's *t* test or Mann–Whitney *U* test was used for normally or not normally distributed data, respectively. Chi-square test was used for comparison of qualitative data between study groups. The predictive value of NLR for tinnitus was evaluated using receiver operating characteristic (ROC) analysis and optimal cut-off point of NLR was determined with either high sensitivity or high specificity. The odds ratio was calculated as an estimate of relative risk of cut-off NLR on the tinnitus as an outcome. Statistical significance was defined as *p* < 0.05.

## Results

Of 544 patients included in the study, 54.2% were female and 45.8% were male. The ratio of female patients was higher in the tinnitus group than the control group (58.5% vs. 41.5%, respectively; *p* = 0.077) (Table 1). The age of study population ranged from 18 to 59 years with a mean of 41.81 ± 11.29 years. The mean age of patients with tinnitus was significantly higher than those of control group (44.89 ± 10.96 years and 38.37 ± 10.65 years, respectively; *p* = 0.001) (Table 1).

**Table 1:** Demographics and concomitant diseases of the study groups.

	Total (*n* = 544)	Patients with tinnitus (*n* = 287)	Healthy controls (*n* = 257)	*p*
*Age (year)*	41.81 ± 11.29	44.89 ± 10.96	38.37 ± 10.65	0.001^a^

*Sex*				0.077^b^
Female	295 (54.2%)	168 (58.5%)	127 (49.4%)	
Male	249 (45.8%)	119 (41.5%)	130 (50.6%)	

*Concomitant diseases*				
Hypertension	74 (13.6%)	56 (19.5%)	18 (7.0%)	0.001^b^
Diabetes mellitus	76 (14.0%)	55 (19.2%)	21 (8.2%)	0.001^b^
Hyperlipidemia	57 (10.5%)	42 (14.6%)	15 (5.8%)	0.001^b^
Psychiatric disorders	53 (9.7%)	32 (11.1%)	21 (8.2%)	0.242^b^
Hypothyroidism	52 (9.6%)	32 (11.1%)	20 (7.8%)	0.182^b^

a Student's *t* test.

b Chi-square test. Data are presented as mean ± standard deviation or *n* (%).

The concomitant diseases recorded in the entire study population were hypertension (13.6%), diabetes mellitus (14.0%), hyperlipidemia (10.5%), psychiatric disorders (9.7%), and hypothyroidism (9.6%) (Table 1). Hypertension, diabetes mellitus, and hyperlipidemia were significantly more common in patients with tinnitus than control group (*p* = 0.001 for all) (Table 1).

There was no statistically significant difference between the groups in terms of pure tone averages of all frequencies (*p* > 0.05).

**Figure 1:**
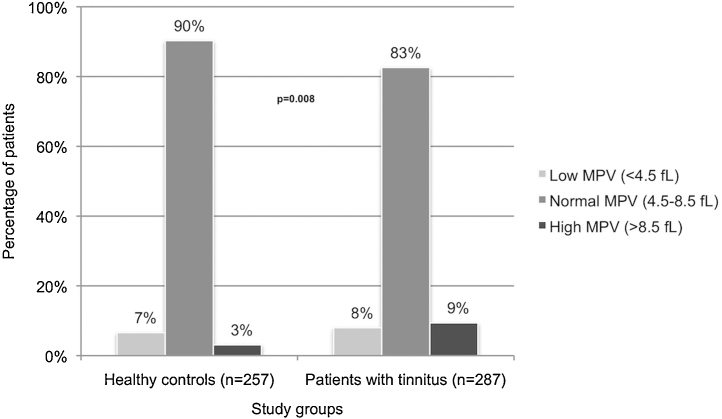
The mean platelet volume (MPV) of healthy controls and patients with tinnitus. As the percentage of subjects with normal MPV level was lower in tinnitus group than the control group, those of low or high MPV level was significantly higher (*p* = 0.008) (fL, femtoliter).

MPV levels were within normal limits in 86.2% of the entire study population. The percentage of subjects with high MPV level was significantly higher in tinnitus group than the control group (9.4% and 3.1%, respectively; *p* = 0.008) (Table 2, Fig. 1).

**Table 2:** Mean platelet volume and neutrophil to lymphocyte ratio of the study groups.

	Total (*n* = 544)	Patients with tinnitus (*n* = 287)	Healthy controls (*n* = 257)	*p*
*MPV*				0.008^a^
Low (<4.5 fL)	40 (7.4%)	23 (8.0%)	17 (6.6%)	
Normal (4.5–8.5 fL)	469 (86.2%)	237 (82.6%)	232 (90.3%)	
High (>8.5 fL)	35 (6.4%)	27 (9.4%)	8 (3.1%)	

*NLR*	1.82 ± 0.85	1.95 ± 1.02	1.67 ± 0.57	0.012^b^
	1.6 (0.6–9.9)	1.7 (0.9–9.9)	1.6 (0.6–3.7)	

a Chi-square test.

b Mann–Whitney *U* test. MPV, mean platelet volume; NLR, neutrophil to lymphocyte ratio; Fl, femtoliter. Data are presented as *n* (%) or mean ± standard deviation (median, range).

The mean NLR of the study population was 1.82 ± 0.85, being higher in patients with tinnitus than control group (1.95 ± 1.02 and 1.67 ± 0.57, *p* = 0.012) (Table 2).

**Figure 2:**
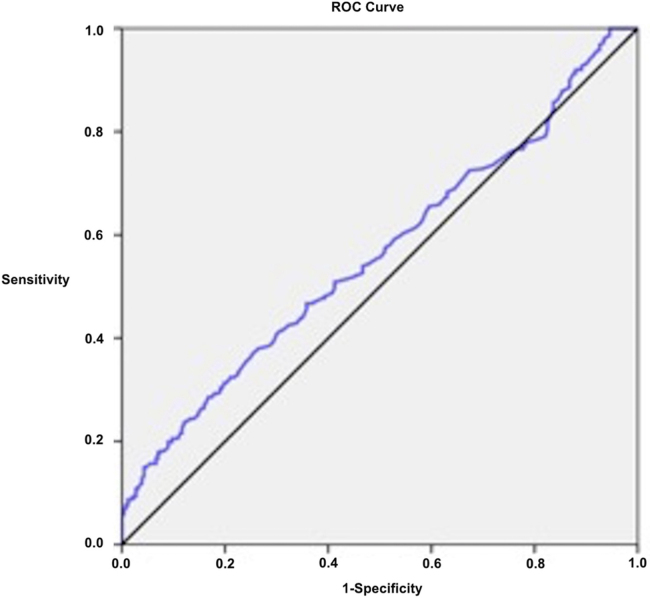
Receiver operating characteristic (ROC) curve for neutrophil to lymphocyte ratio (NLR) to predict tinnitus.

**Figure 3:**
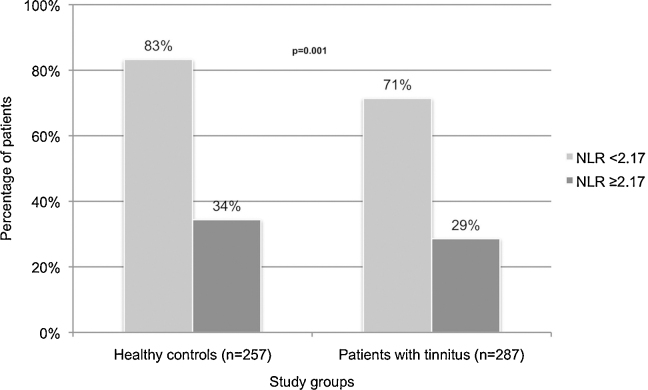
Distribution of individuals in tinnitus and control groups with respect to neutrophil to lymphocyte ratio (NLR) cut-off value of 2.17. The percentage of patients having a NLR of 2.17 and above was 65.6% in the tinnitus group and 34.4% in control group (*p* = 0.001).

The optimal cut-off value of NLR for tinnitus was calculated as 2.17 (sensitivity 28.6%, specificity 83.3%) in ROC analysis (Table 3, Fig. 2). The percentage of patients having a NLR of 2.17 and above was 65.6% in the tinnitus group and 34.4% in control group (*p* = 0.001) (Fig. 3). A NLR level of 2.17 and above may be associated with 1.99 times higher risk of tinnitus (OR = 1.99, 95% CI 1.31–3.02) (Table 3).

**Table 3:** Predictive value of neutrophil to lymphocyte ratio for tinnitus in ROC analysis.

Predictive value of NLR							ROC curve		
Cut-off	Sensitivity	Specificity	PPV	NPV	OR	95% CI	AUC	95% CI	*p*
≥2.17	28.6%	83.3%	65.60	51.07	1.99	1.31–3.02	0.563	0.515–0.611	0.011

NLR, neutrophil to lymphocyte ratio; ROC, receiver operating characteristic; PPV, positive predictive value; NPV, negative predictive value; OR, odds ratio; CI, confidence interval.

## Discussion

In this case-control study, we primarily found that MPV and NLR levels are significantly higher in patients with tinnitus than in healthy individuals. We also calculated a cut-off value of NLR as 2.17, which is associated with 1.991 time's higher risk of tinnitus. Additionally, we recorded that hypertension, diabetes mellitus, and hyperlipidemia were significantly more common in patients with tinnitus.

Tinnitus is most common in the population over 50 years.^14^, ^15^, ^16^ The incidence of tinnitus has been increasing with an increase in progressive age-related hearing loss in aging population.^17^, ^18^, ^19^ In our study, we defined upper age limit as 59 years to exclude patients with presbycusis. Additionally, patients who had early onset bilateral sensorineural hearing loss according to audiometric data were excluded. In most of the previous studies on tinnitus, the individuals with presbyacus have been included without any upper age limit.^17^, ^18^, ^19^ The significantly higher mean age of patients with tinnitus than those of healthy individuals in our study was interpreted as the increase in incidence of tinnitus in later ages. We suggest that even though age-related hearing loss has not developed, tinnitus is a growing complaint in later ages. Therefore, it is necessary to evaluate other etiologic factors that are responsible for the increase in incidence of tinnitus with age.

In previous studies, conflicting results were obtained on gender distribution of tinnitus. Schulman et al.^14^ reported that there was no difference between male and female patients in terms of frequency of tinnitus. In another study, the tinnitus was more common in young male patients than young females, but as the age increases, the rates were equalized between genders.^20^ Martines et al.^15^ found that the frequency of tinnitus significantly increased in male individuals aged 50 years and over. Vernon et al.^21^ reported that tinnitus is more common in males than females in all age groups. In contrary, Kim et al.^16^ found that the rate of female subjects was higher in tinnitus group than control group, indicating that tinnitus is more common among women than men.

Recent studies have shown that hypertension increases the risk of tinnitus.^15^, ^22^, ^23^ Supporting this relation, we found that hypertension was significantly more common in tinnitus group than the control group. Figueiredo et al.^23^ reported that changes in blood pressure may induce tinnitus by affecting cochlear microcirculation, or that tinnitus may result from the use of antihypertensives such as verapamil and enalapril in these patients. In our study, the use of antihypertensives in the study population was not investigated. If patients using antihypertensives were excluded from the study, the relationship between hypertension and tinnitus could be more clearly demonstrated, and the hypothesis that hypertension may disrupt cochlear microcirculation would be tested.

Somogyi et al.^24^ suggested that type 2 diabetes increases the frequency of tinnitus, and tinnitus occurs at an earlier age in patients with type 2 diabetes. In our study, diabetes mellitus was significantly more prevalent in the tinnitus group than the control group, supporting an association between diabetes mellitus and tinnitus. Although patients with age-related hearing loss were excluded from the study by setting an upper age limit, individuals with tinnitus had still a significantly higher age. We think that diabetes mellitus causes tinnitus by affecting cochlear microcirculation, and it may be one of the reasons why tinnitus is more common in our advanced age population.

Martines et al.^15^ found that the frequency of tinnitus was significantly increased in cases with hypercholesterolemia. Similarly, Kim et al.^16^ reported that hyperlipidemia constitutes a risk factor for tinnitus. In parallel to these reports, the rate of hyperlipidemia was significantly higher in patients with tinnitus in than healthy controls in our study.

Kim et al.^16^ suggested that the presence of stress and depression constituted a risk factor for tinnitus. Fetoni et al.^25^ observed that the tinnitus disability questionnaire score was higher as the depression-anxiety depth increased. Ziai et al.^26^ found a significant association between tinnitus and psychiatric disorders. Unlike the majority of studies in the literature, the prevalence of psychiatric disorders in our study did not differ significantly between patients and control groups.

Kim et al.^16^ also found that the thyroid disease was a risk factor for tinnitus. However, hypothyroidism was not associated with tinnitus in our study.

In the previous studies, MPV values of individuals with subjective tinnitus were reported to be higher.^27^, ^28^ In line with these studies, we found that in comparison to healthy controls, high MPV values were significantly more prevalent in tinnitus group. Macrothrombocytes can disturb cochlear circulation because of their high thrombotic potential.^6^ The hairy cell neuronal damage increases spontaneous activity establishing abnormal synapses and sending false information to auditory cortex, which causes the patient to hear a sound.^6^

Özbay et al.^29^ compared the NLR values of individuals with and without tinnitus, and reported that those with tinnitus had significantly higher NLR. Similarly, in our study the tinnitus group had significantly higher NLR values than control group. In ROC analysis, the cut-off value of NLR to predict tinnitus with high specificity was 2.17. NLR is an etiologic and prognostic factor for diseases characterized by microcirculation failure and end organ damage.^30^ Increased NLR in tinnitus suggests that the cochlear microcirculation failure may be the underlying pathology of tinnitus.

The main limitation of the present study was its retrospective design, which creates limits in controlling confounding factors affecting MPV and NLR, and precludes us from reaching a definitive conclusion on vascular pathologies in etiology of tinnitus. Further large-scale, randomized, prospective studies are required to clearly define the etiopathology of tinnitus.

## Conclusion

In conclusion, high MPV and NLR values may be associated with idiopathic tinnitus, suggesting the role of vascular pathologies in the etiology of tinnitus. Although tinnitus is only a symptom, it may indicate underlying systemic disorders. Therefore, patients with tinnitus should undergo detailed systemic examination, medical history, and laboratory tests including hematological indices.

## Financial support

This research did not receive any specific grant from funding agencies in the public, commercial, or not-for-profit sectors.

## Conflicts of interest

The authors declare no conflicts of interest.
